# Bisphenol A Impairs Lipid Remodeling Accompanying Cell Differentiation in the Oligodendroglial Cell Line Oli-Neu

**DOI:** 10.3390/molecules27072274

**Published:** 2022-03-31

**Authors:** Vanessa Naffaa, Isabelle Hochar, Chéryane Lama, Romain Magny, Anne Regazzetti, Pierre Gressens, Olivier Laprévote, Nicolas Auzeil, Anne-Laure Schang

**Affiliations:** 1 CiTCoM, CNRS, Université Paris Cité, 75006 Paris, France; vanessa.naffaa@parisdescartes.fr (V.N.); isabellehochar@hotmail.com (I.H.); cheretidel@hotmail.fr (C.L.); romain.magny@inserm.fr (R.M.); anne.regazzetti@parisdescartes.fr (A.R.); olivier.laprevote@parisdescartes.fr (O.L.); nicolas.auzeil@parisdescartes.fr (N.A.); 2 INSERM UMR 968, CNRS UMR 7210, Institut de la Vision, IHU ForeSight, Sorbonne Université UM80, 75012 Paris, France; 3 NeuroDiderot, Inserm, Université Paris Cité, 75019 Paris, France; pierre.gressens@inserm.fr; 4 Hôpital Européen Georges Pompidou, AP-HP, Service de Biochimie, 75015 Paris, France; 5 UMR 1153 CRESS, Université Paris Cité, 75004 Paris, France

**Keywords:** oligodendrocyte, differentiation, bisphenol A, lipidomics

## Abstract

In the central nervous system, the process of myelination involves oligodendrocytes that wrap myelin around axons. Myelin sheaths are mainly composed of lipids and ensure efficient conduction of action potentials. Oligodendrocyte differentiation is an essential preliminary step to myelination which, in turn, is a key event of neurodevelopment. Bisphenol A (BPA), a ubiquitous endocrine disruptor, is suspected to disrupt this developmental process and may, thus, contribute to several neurodevelopmental disorders. In this study, we assessed the effect of BPA on oligodendrocyte differentiation through a comprehensive analysis of cell lipidome by UHPLC-HRMS. For this purpose, we exposed the oligodendroglial cell line Oli-neu to several BPA concentrations for 72 h of proliferation and another 72 h of differentiation. In unexposed cells, significant changes occurred in lipid distribution during Oli-neu differentiation, including an increase in characteristic myelin lipids, sulfatides, and ethanolamine plasmalogens, and a marked remodeling of phospholipid subclasses and fatty acid contents. Moreover, BPA induced a decrease in sulfatide and phosphatidylinositol plasmalogen contents and modified monounsaturated/polyunsaturated fatty acid relative contents in phospholipids. These effects counteracted the lipid remodeling accompanying differentiation and were confirmed by gene expression changes. Altogether, our results suggest that BPA disrupts lipid remodeling accompanying early oligodendrocyte differentiation.

## 1. Introduction

During development, the brain is exposed to a myriad of endocrine disrupting chemicals (EDCs) which can, even at low doses, interact with multiple cell signaling pathways and disrupt many biological processes [1,2,3]. Thereby, exposure to environmental EDCs is suspected to be involved in the growing incidence of neurodevelopmental disorders. These conditions, which include sensorimotor, cognitive and learning deficits, attention deficit disorders with or without hyperactivity (ADHD), and autism spectrum disorders (ASD), affect more than 10% of children worldwide and constitute a major public health concern [4]. According to the WHO, the incidence of neurodevelopmental disorders has dramatically increased over the past decades. For example, the incidence of ASD, estimated at 4 or 5 cases per 10,000 children in the 1970s, was reassessed at 1 per 110 in the 2000s [5]. As these pathologies are complex and multifactorial, the contribution of EDCs is difficult to demonstrate and quantify. In humans, the first correlations have been established between in utero exposure to organophosphate pesticides and ADHD prevalence, and between exposure to polychlorinated biphenyls and reduced intellectual capacity [5]. Recently, several studies have correlated exposure to bisphenol A (BPA) with increased cognitive and behavioral disorders [6,7]. BPA has been extensively used in industry, mainly to produce polycarbonate plastic and epoxy resins. Despite restriction of its use adopted in many countries, BPA is still ubiquitous in the environment and, consequently, in human biological fluids. In 2017, BPA was classified among the “substances of very high concern” in the REACH regulation [8].

Although EDCs are likely to be involved in the etiology of neurodevelopmental disorders, so far, their toxicity has been poorly evaluated in the immature brain [9]. In fact, in epidemiological and in vivo studies, intellectual, cognitive, emotional, and social behaviors are difficult to assess and quantify, making causal relationships difficult to establish. Thereby, it seems relevant to identify cellular and molecular mechanisms of EDC toxicity to define target processes and molecular markers that could ultimately allow a better evaluation of these compounds. Given the central role of neurons in the central nervous system (CNS) function and the fact that neuronal connectivity is impaired in many neurodevelopmental disorders, most studies have focused on EDCs’ impacts towards neurogenesis and synaptic plasticity [9]. Conversely, few studies have analyzed the impact of EDCs on glial cells, and in particular, on oligodendrocytes, even though they carry out myelination, a fundamental step of CNS development [9]. These cells arise later than neurons, from the third trimester of pregnancy. During this period, oligodendrocyte precursor cells (OPCs) proliferate, migrate, and differentiate into immature, and then mature, oligodendrocytes which myelinate the CNS by wrapping myelin sheaths around axons. In addition to their protective role, myelin sheaths increase the propagation rate of action potentials by insulating nerve fibers. Abnormal myelination contributes to neurological deficits. In particular, white matter alterations have been associated with ADHD and ASD [10,11]. By disrupting myelination, EDCs could contribute to the development of such disorders. Indeed, some studies have indicated that EDCs, especially BPA, can disrupt oligodendrocyte differentiation and myelination, but the evidence is still scarce [3]. It must be emphasized that lipids represent 70% of myelin dry weight and, therefore, are, by far, the main myelin constituents [12]. Thus, lipids are a valuable molecular target to assess oligodendrocyte differentiation and its disruption.

**Figure 1:**
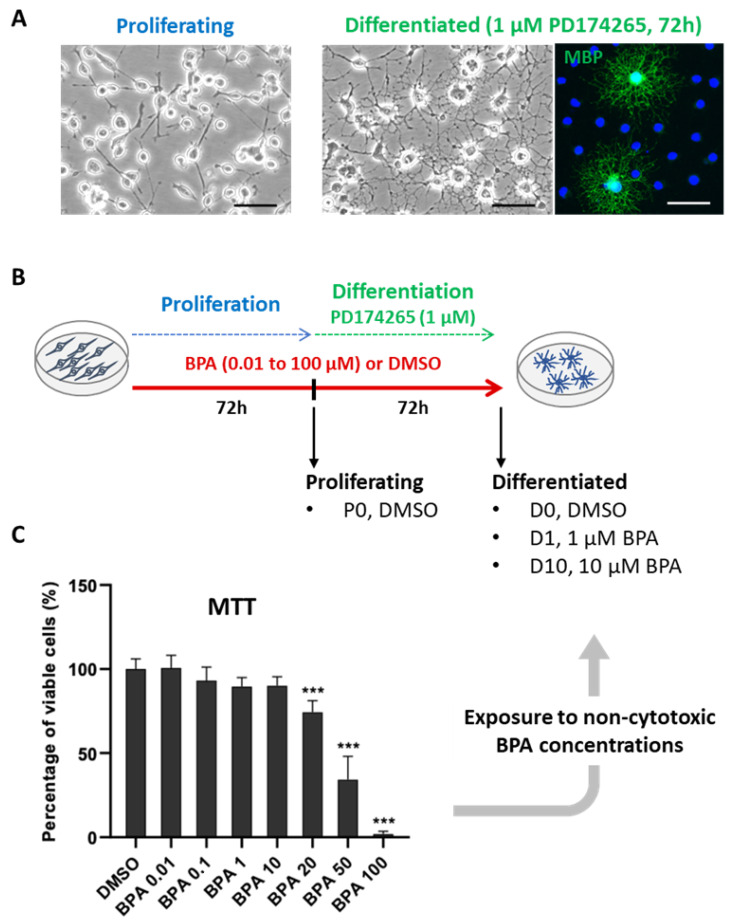
Oli-neu morphology, protocol of exposure to BPA and viability assay: ((**A**), **left panel**) Proliferating Oli-neu cells displaying bipolar morphology; ((**A**), **right panel**) Oli-neu cells displaying ramifications and positive MBP immunostaining in a subset of cells after 72 h of differentiation induced by pharmacological agent PD174265 (1 µM), scale bars 50 µM; (**B**) protocol of exposure to bisphenol A (BPA) for 72 h of proliferation and 72 h of differentiation. P0— proliferating cells, D0—differentiated control cells, D1—differentiated cells exposed to 1 µM BPA, D10—differentiated cells exposed to 10 µM BPA; (**C**) cell viability determined by MTT assay (*N* = 5), results are expressed as mean ± SD percentage of viable cells as compared with the DMSO control (one-way ANOVA and Dunnett multiple comparison post-test, *** *p* < 0.001).

The aim of the present study was to assess the impact of BPA on oligodendrocyte differentiation in vitro through a comprehensive analysis of cellular lipid contents. For this purpose, we used the oligodendroglial cell line Oli-neu. Oli-neu are immortalized mouse OPCs transformed with activated *ErbB2* gene [13]. These cells can undergo differentiation especially using the pharmacological agent PD174265 [14,15]. In proliferating conditions, Oli-neu cells exhibit a bipolar morphology typical of OPCs, while during differentiation, they become increasingly ramified and display an immature oligodendrocyte shape after 72 h (Figure 1A). Moreover, proliferating Oli-neu cells express several markers of late OPCs including O4 and 2′,3′-cyclic nucleotide 3′ phosphodiesterase (CNPase) [15]. During differentiation, CNPase and markers of mature oligodendrocytes accumulate, including myelin basic protein (MBP) (Figure 1A) [15]. Therefore, the Oli-neu cell line was chosen to model early oligodendrocyte differentiation. To investigate the impact of BPA on the lipidome of differentiating Oli-neu, the cells were exposed to several non-cytotoxic BPA concentrations for 72 h before the onset of differentiation and another 72 h during differentiation (Figure 1B). Changes in lipidome accompanying Oli-neu differentiation were primarily characterized. As expected, Oli-neu differentiation led to a clear enrichment in several characteristic myelin lipid subclasses including ethanolamine plasmalogens (ePE) and sulfatides (ST). Importantly, the increase in ST was impaired by BPA, which could indicate a direct effect of this compound on oligodendrocyte early differentiation.

## 2. Material and Methods

### 2.1. Oli-Neu Culture, Differentiation, and BPA Treatment

Oli-neu cells were maintained in DMEM, high glucose GlutaMAX^TM^ Supplement, pyruvate (Gibco reference 31966047, Thermo Fisher Scientific, Courtaboeuf, France) supplemented with 1% N2 (Gibco reference 17502001, Thermo Fisher Scientific, Courtaboeuf, France), 1% heat inactivated horse serum (Gibco reference 26050070, Thermo Fisher Scientific, Courtaboeuf, France), 1% penicillin-streptomycin (10,000 U/mL) (Gibco reference 15140122, Thermo Fisher Scientific, Courtaboeuf, France), 10 µg/mL insulin from bovine pancreas (Sigma I5500, Saint Quentin Fallavier, France), 0.4 µg/mL L-Thyroxine sodium salt pentahydrate (T4) (Sigma T0397, Saint Quentin Fallavier, France,), and 0.34 µg/mL 3,3′,5-Triiodo-L-thyronine sodium salt (T3) (Sigma T6397 Saint Quentin Fallavier, France).

Flasks and wells were coated with poly-L-lysine (Sigma P4707, Saint Quentin Fallavier, France) diluted at 20 µg/mL. Cells were exposed to BPA (Sigma 239658, Saint Quentin Fallavier, France) during 72 h of proliferation and an additional 72 h of differentiation induced by 1 μM PD174265 in DMSO (Sigma 513040-M, Saint Quentin Fallavier, France). The medium was changed after 48 h of differentiation. Oli-neu cells were exposed to 0.01, 0.1, 1, 10, 20, 50, and 100 µM BPA in DMSO. The final concentration of DMSO was 0.2%.

### 2.2. Cell Viability

Oli-neu cells were treated with BPA (0.01, 0.1, 1, 10, 20, 50, and 100 μM) in triplicate, in 96-well plates, during proliferation (72 h) followed by differentiation (72 h). Thiazolyl blue tetrazolium bromide (MTT, Merck M5655, Saint Quentin Fallavier, France) was extemporaneously diluted to prepare a 0.5 mg/mL solution in culture medium. At the end of the BPA treatment, 100 µL of MTT solution was directly added to the medium culture and cells were incubated for 3 h at 37 °C. After incubation, the medium was removed, cells were lysed with DMSO (100 μL), and homogenized (RT, 15 min) on a shaker plate. The optical density was read at 570 nm on a SpectraMax paradigm spectrophotometer (Molecular devices, Villepinte, France). The means were calculated from 5 independent experiments.

### 2.3. Western Blotting

Frozen cell pellets were homogenized in cold RIPA buffer (Sigma R0278, Saint Quentin Fallavier, France) containing protease inhibitors (cOmplete^TM^ Tablets, Roche 4693159001, Merck, Saint Quentin Fallavier, France) for 1 h. An equal amount of 10 µg protein per sample was used. Western Blot migration, transfer, incubation, revelation, and quantification were performed as previously described using mouse anti-β-actin (1:20,000, Sigma A5316, Saint Quentin Fallavier, France), mouse anti-CNPase (1:500, Merck-Millipore MAB326, Guyancourt, France), and HRP-conjugate goat anti-mouse IgG (1:2000, Sigma 12-349, Saint Quentin Fallavier, France) antibodies [16]. Actin was used as loading control.

### 2.4. Immunocytofluorescence

Oli-neu cells were plated on poly-L-lysine-coated Ibidi 8-well chamber slides (BioValley, Marne la Vallee, France) and exposed to BPA. Then, cells were fixed with 4% paraformaldehyde, permeabilized with 0.1% triton and saturated with 3.5% donkey serum in PBS. Immunocytofluorescence staining was performed using mouse anti-MBP (1:200, Merck-Millipore MAB382, Guyancourt, France) and mouse anti-CNPase (1:250, Merck-Millipore MAB326, Guyancourt, France). Cells were incubated overnight with primary antibodies at 4 °C, washed three times, and incubated with donkey anti-mouse secondary antibody conjugated to Alexa fluor 488 (1:500, Invitrogen A21202, Villebon-sur-Yvette, France) for 1 h. DAPI was used to counterstain nuclei (Sigma D9542, Saint Quentin Fallavier, France). Images were acquired using an Eclipse Ti-E inverted fluorescence microscope and the NIS-Elements software (Nikon, Champigny sur Marne, France).

### 2.5. RT-PCR

Cells (*N* = 6 to 9 independent experiments per group) were lysed and total RNA was extracted with a Nucleospin RNA XS Plus kit (Macherey Nagel, Hoerdt, France). Then, RNA was dosed with a Qubit broad range kit using QFX DeNovix Fluorometer (Proteigene, Saint-Marcel, France), and 1 µg RNA was subjected to retrotranscription using an iScript^TM^ cDNA synthesis kit (Bio-Rad, Marnes-La-Coquette, France). Then, qPCRs were achieved for each sample in duplicates using SYBR^®^ Green Supermix (Bio-Rad, Marnes-La-Coquette, France) in a CFX96 Real-Time PCR machine (Bio-Rad, Marnes-La-Coquette, France), with a 3-step program (5 s of denaturation at 95 °C, 10 s of annealing at 60 °C, and 10 s of elongation at 72 °C). Specific primers (Eurogentec, Angers, France) were designed with the NCBI primer design tool. Sequences are given in Supplementary Table S1. *Rpl13a* (ribosomal protein L13a) was chosen to normalize the quantitative experiments based on reference gene suitability testing. The results are expressed as relative expression to D0 samples.

### 2.6. Lipidomic Analysis

#### 2.6.1. Cell Lysis and Lipid Extraction

Cell pellets of 2.5 million cells (*N* = 6 independent experiments per group) were stored at −80 °C until the start of the analytical process. Pellets were resuspended in 1 mL of ultra-pure water and sonicated for 5 min. Then, suspensions were spiked with 15 µL of a mixture of internal standards (15 µM concentration each) and extracted with 7.25 mL of a chloroform/methanol/water mixture (2:2:1.8 *v*/*v*/*v*) containing 3,5-di-tert-4-butylhydroxytoluene 0.01% (*w*/*v*) as antioxidant agent. Then, samples were centrifuged at 3000 rpm for 10 min. Organic phases were collected, evaporated to dryness at 45 °C under reduced pressure, and the lipid extracts were dissolved in 90 µL of acetonitrile/isopropanol/chloroform/water (35:35:20:10) mixture.

#### 2.6.2. Sample Injection and Data Analysis

Sample injection was achieved using an ultra-high-performance liquid chromatography high-resolution mass spectrometry (UHPLC-HRMS) device consisting of a UPLC^®^ system (Waters, Manchester, UK) hyphenated to a Synapt^®^ G2 (Q-TOF) mass spectrometer (Waters, Manchester, UK). Chromatographic separation was achieved on an Acquity^®^ CSH C18 column (100 mm × 2.1 mm, 1.7 μm). UHPLC-HRMS data acquisition and analysis were adapted from a previously described protocol [17]. Data dependent acquisition was performed to provide tandem mass spectra of the five most intense ions detected on a first mass spectrum. Lipid annotation included polar head group identification in glycerophospholipids, sphingoid base characterization in sphingolipids, and fatty acyl side chains. Moreover, *sn*-1 and *sn*-2 locations of fatty acid side chains in phospholipids were determined based on the relative intensity of carboxylate product ions displayed on the MS/MS spectra. The regioisomer mentioned for each lipid species identified corresponded to the major regioisomer (Supplementary Table S2). However, the presence of a minor amount of the other regioisomer cannot be excluded. Concentration of each lipid species was determined using the corresponding internal standard. Lipid content was expressed as percentage of total fatty acid (mol%), which grants the advantage of comparing lipid distribution between samples without bias from their individual total lipid amount. The heatmap was built using statistical analysis (one factor) in Metaboanalyst 5.0 software (Montreal, Canada) with the default parameters [18].

### 2.7. Statistical Analysis

Principal component analysis score plots were generated using the SIMCA-P+ software version 13.0.3 (Umetrics, Umeå, Sweden). Statistical analyses such as *t*-tests were performed on GraphPad Prism 8.0 (Graph-Pad Software, San Diego, CA, USA). Mol% of lipid subclasses and species were compared by a multiple *t*-test. MTT viability and gene expression data were analyzed by one-way ANOVA and Dunnett multiple comparison post-test after validation of normal distribution by a Shapiro–Wilk test. D0 was used as control group. The value of *p* < 0.05 was considered to be statistically significant.

## 3. Results

A MTT viability assay was achieved by exposing Oli-neu cells to BPA concentrations ranging from 0 to 100 µM during 72 h of proliferation followed by 72 h of differentiation (Figure 1C). Cytotoxicity appeared from 20 µM BPA concentration (74.3 ± 6.1% viable cells, *p* < 0.001), and cell viability gradually decreased to 1.9 ± 1.4% viable cells (*p* < 0.001) at 100 µM of BPA. The subcytotoxic doses of BPA (up to 10 µM) were, thus, selected to perform lipidomic and gene expression analyses in Oli-neu cells (Figure 1B). Four groups were finally considered: proliferating cells (P0), differentiated control cells (D0), differentiated cells exposed to BPA 1 µM (D1), and differentiated cells exposed to BPA 10 µM (D10).

**Figure 2:**
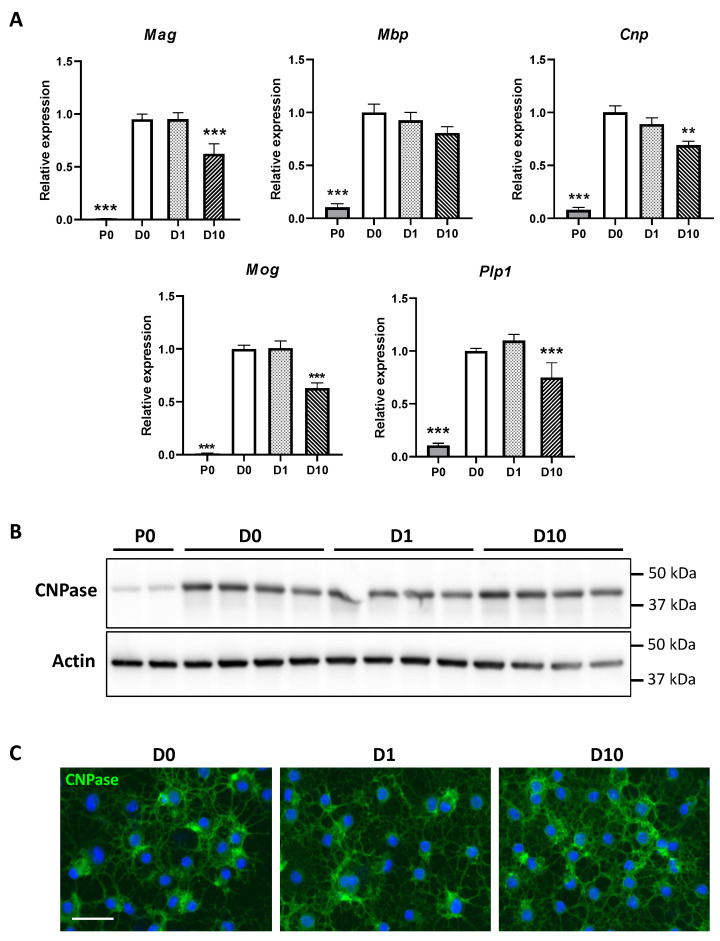
Myelin gene expression and CNPase immunoreactivity in Oli-neu cells: (**A**) Gene expression analysis of myelin proteins: myelin basic protein (*Mbp*), myelin-associated glycoprotein (*Mag*), 2′,3′-cyclic nucleotide 3′ phosphodiesterase (*Cnp*), myelin oligodendrocyte glycoprotein (*Mog*), and proteolipid protein 1 (*Plp1*), (*N* = 6–9), relative expression was normalized to reference gene *Rpl13a*, all groups were compared to D0 control group, (one-way ANOVA and Dunnett multiple comparison post-test, ** *p* < 0.01, and *** *p* < 0.001); (**B**) Western blot of CNPase and actin; (**C**) CNPase staining by immunocytofluorescence, with DAPI counterstaining. Scale bar, 50 µM. P0—proliferating cells, D0—differentiated control cells, D1—differentiated cells exposed to BPA 1 µM, D10—differentiated cells exposed to BPA 10 µM.

To validate the differentiation protocol and address the effects of BPA through well-known markers of oligodendrocyte differentiation, we first assessed the expression of five genes coding myelin proteins, i.e., myelin basic protein (*Mbp*), myelin-associated glycoprotein (*Mag*), 2′,3′-cyclic nucleotide 3′ phosphodiesterase (*Cnp*), myelin oligodendrocyte glycoprotein (*Mog*), and proteolipid protein 1 (*Plp1*) (Figure 2A). Expression of these myelin genes experienced a dramatic increase between P0 and D0. *Plp1*, *Mbp*, *Cnp*, *Mog*, and *Mag* expressions increased by 9.4 (*p* < 0.001), 9.7 (*p* < 0.001), 12.5 (*p* < 0.001), 98.0 (*p* < 0.001), and 104.2 folds (*p* < 0.001), respectively. In differentiated Oli-neu, BPA exposure at 10 µM decreased the expression of *Plp1*, *Cnp*, *Mog*, and *Mag* genes by 0.75 (*p* < 0.001), 0.69 (*p* < 0.01), 0.63 (*p* < 0.001), and 0.62 (*p* < 0.001), respectively. Moreover, we investigated the levels of CNPase protein, an early expressed myelin-associated enzyme [15]. Western blot analysis revealed low levels of CNPase in proliferating cells, while a strong CNPase signal was evidenced in differentiated cells, with comparable intensity among D0, D1 and D10 (Figure 2B). In addition, CNPase was clearly detected by immunocytofluorescence in differentiated cells (Figure 2C). As observed in Western blot, the intensity of CNPase staining appeared similar between control D0 cells and cells exposed to BPA. Finally, on the one hand, the efficiency of differentiation was confirmed by the upregulated expression of several genes coding for myelin proteins and CNPase accumulation. On the other hand, 10 µM BPA partially counteracted the increase in the expression of several genes coding for myelin proteins, including *Cnp*, without affecting CNPase immunoreactivity.

**Figure 3:**
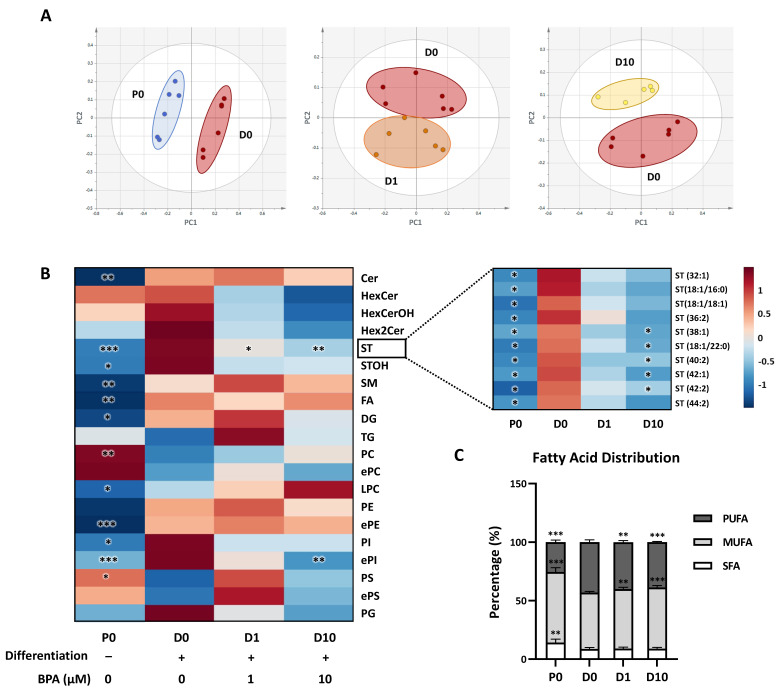
Lipid distribution of Oli-neu cells subjected to differentiation and BPA exposure: (**A**) Principal component analysis of proliferation (P0) and differentiated groups (D0, D1, and D10); (**B**) heatmap representing all studied lipid subclasses in proliferation (P0) and differentiation (D0, D1, and D10) groups (*N* = 6), created using the MetaboAnalyst 5.0 Software default parameters and Supplementary Table S4; (**C**) stacked bars representing percentages of fatty acid side chain distribution in phospholipids. P0—proliferating cells, D0—differentiated control cells, D1—differentiated cells exposed to BPA 1 µM, D10—differentiated cells exposed to BPA 10 µM, SFA—saturated fatty acid, MUFA—monounsaturated fatty acid, PUFA—polyunsaturated fatty acid. All groups were compared to D0 control group and expressed in percentages, as mean ± SD (multiple *t*-test, * *p* < 0.05, ** *p* < 0.01, and *** *p* < 0.001).

A comprehensive lipidomic analysis was then performed by UHPLC-HRMS to assess changes accompanying Oli-neu differentiation and the impacts of BPA. Principal component analysis (PCA) models comparing lipid distribution (mol%) of P0, D0, D1, and D10 showed clear separation between P0 and a cluster including D0, D1, and D10 (Supplementary Figure S1). Nevertheless, a clear clustering was observed when comparing D0 to P0, D0 to D1, and D0 to D10 (Figure 3A). It is noteworthy that no separation was exhibited between D0 and differentiated cells exposed to lower concentrations of BPA, 0.01 µM and 0.1 µM (data not shown).

Regarding differentiation of unexposed cells, comparison of P0 to D0 revealed significant lipid distribution changes (Figure 3B, Supplementary Tables S3 and S4). A marked increase was observed from P0 to D0 for both phosphatidylethanolamine (PE) and their ether analogs (ePE), respectively, from 3.1 ± 0.27 mol% to 4.8 ± 0.21 mol% and from 3.1 ± 0.23 mol% to 7.3 ± 0.72 mol% (*p* < 0.001). Regarding phospholipids, additional changes included an increase in phosphatidylinositol (PI) content from 5.3 ± 1.7 mol% to 10.0 ± 3.3 mol% (*p* < 0.05). It was concomitant to a dramatic decrease in phosphatidylcholine (PC) abundance (from 72.0 ± 4.8 mol% to 59.6 ± 5.1 mol%, *p* < 0.01) and, to a lesser extent, in phosphatidylserine (PS) (from 0.89 ± 0.17 mol% to 0.62 ± 0.13 mol%, *p* < 0.05). Regarding sphingolipids, differentiation was accompanied by an increase in ceramide (Cer), sulfatide (ST), hydroxylated sulfatide (STOH), and sphingomyelin (SM) amounts, respectively, from 0.13 ± 0.02 mol% to 0.20 ± 0.03 mol% (*p* < 0.01), from 0.46 ± 0.09 mol% to 1.69 ± 0.38 mol% (*p* < 0.001), from 0.09 ± 0.03 mol% to 0.29 ± 0.16 mol% (*p* < 0.05), and from 1.9 ± 0.32 mol% to 2.6 ± 0.17 mol% (*p* < 0.01). Moreover, diglyceride (DG), free fatty acid (FA), and lyso-phosphatidylcholine (LPC) contents also increased between P0 and D0, from 0.20 ± 0.02 mol% to 0.25 ± 0.03 mol% (*p* < 0.05), from 1.7 ± 0.33 mol% to 2.4 ± 0.45 mol% (*p* < 0.01), and from 0.35 ± 0.08 mol% to 0.47 ± 0.09 mol% (*p* < 0.05), respectively. The global composition in fatty acyl side chains was also determined in lipid subclasses (Supplementary Table S2). In phospholipids, from P0 to D0, the global content in saturated FA (SFA) and monounsaturated FA (MUFA) decreased from 14.2 ± 2.9% to 8.6 ± 1.3% (*p* < 0.01) and from 60.4 ± 3.6% to 48.3 ± 1.1% (*p* < 0.001), respectively (Figure 3C). Conversely, the content in polyunsaturated FA (PUFA) increased from 25.4 ± 1.8% in P0 to 43.1 ± 2.1% in D0 (*p* < 0.001).

We next assessed the impact of BPA exposure on Oli-neu differentiation based on lipidomic analysis data. At both 1 and 10 μM doses, BPA decreased ST levels from 1.69 ± 0.38 mol% in D0 to 0.97 ± 0.13 (*p* < 0.05) in D1 and 0.77 ± 0.09 (*p* < 0.01) in D10 (Figure 3B). Among the 10 identified ST species, which were all upregulated during differentiation (*p* < 0.05), 5 species were significantly decreased by 10 µM BPA in differentiated cells (Figure 3B, right panel). A decrease in ePI content was also measured in differentiated cells following exposure to 10 µM BPA (from 0.44 ± 0.08 mol% in D0 to 0.23 ± 0.02 mol% in D10, *p* < 0.001). Regarding the global content of FA side chains in phospholipids, MUFA content was increased from 48.3 ± 1.1% in D0 to 51.0 ± 1.4% in D1 (*p* < 0.01) and 52.3 ± 1.6% in D10 (*p* < 0.001), while PUFA content was decreased from 43.1 ± 2.1% in D0 to 40.0 ± 1.4% in D1 (*p* < 0.01) and 38.7 ± 0.75% in D10 (*p* < 0.001) (Figure 3C).

**Figure 4:**
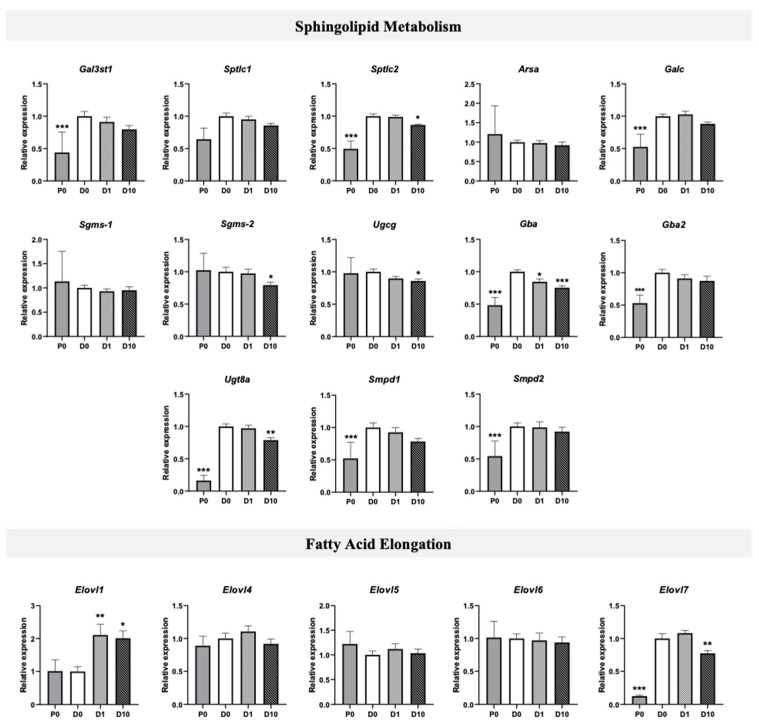
Gene expression of enzymes of lipid metabolism in Oli-neu cells: Gene expression analysis of sphingolipid metabolism (upper panel) and fatty acid elongation (lower panel): cerebroside sulfotransferase enzyme (*Gal3st1*), serine palmitoyltransferase long chain base subunit 1 (*Sptlc1*), serine palmitoyltransferase long chain base subunit 2 (*Sptlc2*), arylsulfatase A (*Arsa*), galactosylceramidase (*Galc*), sphingomyelin synthase 1 (*Sgms1*), sphingomyelin synthase 2 (*Sgms2*), UDP-glucose ceramide glucosyltransferase (*Ugcg*), glucosylceramidase beta (*Gba*), glucosylceramidase beta 2 (*Gba2*), UDP-galactosyltransferase (*Ugt8a*), sphingomyelin phosphodiesterase 1 (*Smpd1*), sphingomyelin phosphodiesterase 2 (*Smpd2*), elongases 1 to 7 (*Elovl1* to *7*). P0—proliferating cells, D0—differentiated control cells, D1—differentiated cells exposed to BPA 1 µM, D10—differentiated cells exposed to BPA 10 µM. *N* = 6–9, relative expression was normalized to reference gene *Rpl13a*, all groups were compared to D0 control group, (one-way ANOVA and Dunnett multiple comparison post-test, * *p* < 0.05, ** *p* < 0.01, and *** *p* < 0.001).

**Figure 5:**
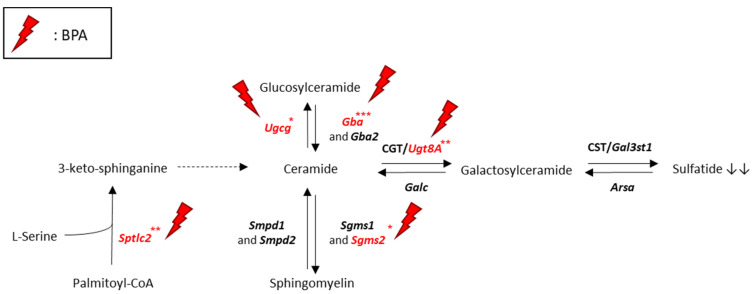
Impact of BPA on sphingolipid metabolism. Adapted from [19,20]: Genes impacted by exposure to BPA 10 µM according to RT-qPCR analysis: serine palmitoyltransferase long chain base subunit 2 (*Sptlc2*), arylsulfatase A (*Arsa*), galactosylceramidase (*Galc*), sphingomyelin synthase 1 (*Sgms1*), sphingomyelin synthase 2 (*Sgms2*), UDP-glucose ceramide glucosyltransferase (*Ugcg*), glucosylceramidase beta (*Gba*), glucosylceramidase beta 2 (*Gba2*), UDP-galactosyltransferase (*Ugt8a*), sphingomyelin phosphodiesterase 1 (*Smpd1*), sphingomyelin phosphodiesterase 2 (*Smpd2*) (one-way ANOVA and Dunnett multiple comparison post-test, * *p* < 0.05, ** *p* < 0.01, and *** *p* < 0.001).

In addition to a lipidome analysis, we investigated lipid metabolism through gene expression by RT-qPCR. The lipidomic analysis revealing changes in lipid contents during Oli-neu differentiation and following BPA exposure, therefore, we assessed the gene expression of enzymes involved in corresponding lipid metabolic pathways (Figure 4). Comparison of lipid contents both between P0 and D0 and between D0 and D1 or D10 groups especially highlighted changes in ST, thus, leading to explore the sphingolipid metabolism pathway (Figure 4 and Figure 5). Between P0 and D0, the expressions of *Gal3st1* coding for cerebroside sulfotransferase enzyme (CST), *Sptlc2* coding for serine palmitoyltransferase long chain base subunit 2 (SPTLC2), and *Ugt8a* coding for ceramide UDP-galactosyltransferase (CGT) exhibited a respective fold change of 2.3 (*p* < 0.001), 2.0 (*p* < 0.001), and 6.2 (*p* < 0.001) (Figure 4). Gene expression of enzymes involved in sphingolipid catabolism, including *Galc* coding for galactosylceramidase (GALC), *Gba* coding for glucosylceramidase beta (GBA), *Gba2* coding for glucosylceramidase beta 2 (GBA2), *Smpd1* coding for sphingomyelin phosphodiesterase 1 (SMPD1), and *Smpd2* coding for sphingomyelin phosphodiesterase 2 (SMPD2), were approximately doubled between P0 and D0 (*p* < 0.001). In differentiated Oli-neu, BPA altered the expression of several genes coding for enzymes of the sphingolipid metabolism pathway (Figure 4 and Figure 5). Among genes exhibiting an increased expression between P0 and D0, BPA induced a decrease in *Sptlc2* and *Ugt8a* expressions, respectively, by 0.86 (*p* < 0.05) and 0.78 (*p* < 0.01) at 10 µM dose (D10) as compared with D0. *Gba* expression, also increased during differentiation, was decreased by BPA exposure in both D1 (0.84, *p* < 0.05) and D10 (0.75, *p* < 0.001) groups. In addition, *Sgms2* coding for sphingomyelin synthase 2 (SGMS2) and *Ugcg* coding for UDP-glucose ceramide glucosyltransferase (UGCG) were decreased in the D10 group by 0.79 (*p* < 0.05) and 0.85 (*p* < 0.05), respectively.

Since SFA, MUFA, and PUFA phospholipid contents also displayed changes between P0 and D0 and between D0 and D1 or D10 groups, fatty acid elongation was investigated through a gene expression analysis of *Elovl* genes coding for elongase enzymes ELOVL1 to ELOVL7 (Figure 4). Expression of *Elovl7* increased by 8-fold (*p* < 0.001) between P0 and D0 groups, whereas *Elovl1*, *Elovl4*, *Elovl5*, and *Elovl6* expressions were not modified. In differentiated Oli-neu, BPA exposure at 10 µM (D10 group) decreased the expression of *Elovl7* by 0.77 (*p* < 0.01). Conversely, the expression of *Elovl1* was increased in both D1 and D10 groups by 2.1- (*p* < 0.01) and 2.0-fold (*p* < 0.05), respectively. (one-way ANOVA and Dunnett multiple comparison post-test, * *p* < 0.05, ** *p* < 0.01, and *** *p* < 0.001).

## 4. Discussion

Oli-neu cells are an OPC immortalized cell line obtained by transformation of mouse OPC with the *t-neu* oncogene. In Oli-neu cells, differentiation induced by PD174265 leads to the acquisition of several hallmarks of immature oligodendrocytes [14]. Oli-neu may, thus, be regarded as a valuable model to assess the toxicological impact of BPA on early oligodendrocyte differentiation. In the present study, this assessment was based on a cell lipidome investigation. A lipidomic analysis was performed by UHPLC-HRMS; this analytical approach made it possible to characterize comprehensively and accurately Oli-neu lipidome. In addition to previous studies including morphological, proteomics, and transcriptomics investigations, our study shows that cell lipidome analysis is a valuable contribution to study oligodendrocyte differentiation [14,15].

BPA is a ubiquitous environmental contaminant that freely crosses the placental barrier and reaches the developing brain throughout fetal life [21]. Exposure to BPA is, therefore, continuous and may occur far before oligodendrocyte differentiation, a late process in brain development that initiates myelination. Thus, we exposed Oli-neu to BPA for 72 h of proliferation and 72 h of differentiation. In addition to the period and duration of exposure, BPA concentration was an essential parameter. Based on a preliminary study investigating BPA cytotoxicity, we assessed the impact of subcytotoxic concentrations ranging from 10 nM to 10 µM on Oli-neu differentiation (Figure 1). In the nanomolar range, BPA did not alter Oli-neu differentiation (data not shown). In contrast, the lipidomic and gene expression analysis highlighted that BPA 1 µM and 10 µM disrupted lipid metabolism during differentiation (Figure 3, Figure 4 and Figure 5). In previous studies, similar BPA concentrations were reported to alter OPC proliferation and differentiation in primary OPCs [22].

### 4.1. Important Lipid Remodeling Occurs during Differentiation in Oli-Neu Cell Line

As previously reported, Oli-neu differentiation with 1 µM PD174265 triggered characteristic morphological changes, CNPase accumulation, and a striking increase in the expression of several genes coding for characteristic myelin proteins, altogether supporting efficient differentiation (Figure 1A and Figure 2) [14,15]. To assess if differentiation induced changes in Oli-neu lipid distribution, we compared the lipidome of differentiated cells to that of undifferentiated. i.e., proliferating cells. Differentiation led to a marked remodeling of Oli-neu lipidome, involving most lipid subclasses. These results especially highlighted important redistribution within phospholipid subclasses including a dramatic increase in PE, ePE, PI, and ePI contents concomitant with a decrease in PC and PS contents. In addition, a systematic identification of phospholipid fatty acyl side chains revealed that differentiation was associated with an enrichment in PUFA, whereas SFA and MUFA contents were decreased. Among other lipid subclasses, an increase was observed in sphingolipid contents, including ST, STOH, Cer, and SM species.

It is noteworthy that ePE and ST are typical lipid subclasses of myelin, since their accumulation is closely associated with myelin biogenesis [12,19]. Such an increase in ePE and ST contents was, therefore, expected in differentiating Oli-neu. Indeed, one may anticipate that during oligodendrocyte differentiation, lipid content of plasma membrane gradually evolves towards that of myelin. Hexosylceramides (HexCer) are other characteristic lipid species of myelin. In contrast to ePE and ST, HexCer relative abundance was mostly unchanged between proliferating and differentiated Oli-neu (Figure 3 and Supplementary Table S2). The lack of HexCer accumulation may originate from differences between native oligodendrocytes and the Oli-neu cell line, or between in vivo and in vitro cellular environments. It may also be hypothesized that HexCer accumulation is delayed as compared with ePE and ST and occurs beyond 72 h of differentiation. Altogether, in view of the lipid remodeling that occurs following PD174265 treatment, the Oli-neu cell line appears to be an appropriate model to investigate oligodendrocyte differentiation, at least at an early stage. To assess the impact of BPA on differentiation, we, thus, characterized the Oli-neu lipidome after BPA exposure.

### 4.2. BPA Disrupted Lipid Remodeling Accompanying Oli-Neu Differentiation

It must be emphasized that while Oli-neu differentiation was characterized by a strong increase of genes coding for myelin proteins, 10 µM BPA reduced the expression of *Plp1*, *Cnp*, *Mog*, and *Mag* (Figure 2A). The lipidome analysis displayed similar trends in ST and ePI amounts which were both decreased by BPA (Figure 3B). Accordingly, BPA decreased the expression of several genes involved in ST metabolism, including *Sptlc2*, *Sgms2*, *Ugcg*, *Ugt8a*, and *Gba* (Figure 4 and Figure 5). Among these genes, *Ugt8a* codes for CGT, an enzyme essential to myelination [23]. Altered *Ugt8a* expression and CGT activity have been associated with myelin disorders induced by toxicants such as lead and methylmercury [3,24,25]. Our results suggest that BPA impaired ST accumulation by modulating sphingolipid metabolic pathways through changes in enzyme expression. This is of great importance since ST are involved in myelin stabilization and maintenance throughout life [19].

BPA also induced changes in the global distribution of FA content in phospholipids by increasing MUFA and decreasing PUFA relative proportions. Indeed, BPA partially prevented FA remodeling associated with differentiation (Figure 3C). This effect, observed at 1 µM, was slightly amplified at 10 µM. Fatty acid synthesis is mediated by several elongases. Expression of *Elovl1* was increased in differentiated Oli-neu exposed to BPA at 1 and 10 µM. Since ELOVL1 is selectively involved in the elongation of SFA and MUFA but not PUFA, especially those with a very long chain, its upregulation by BPA could, therefore, contribute to increase the MUFA content in phospholipids [26]. It is noteworthy that *Elovl1* expression was not changed during differentiation, indicating that the impact of BPA on *Elovl1* expression may not be associated with the differentiation process itself. ELOVL7 is another elongase involved in SFA, MUFA, and, to a lesser extent, PUFA elongation of 18:3 (*n*-3) [27,28]. In differentiated Oli-neu, *Elovl7* expression was decreased at 10 µM BPA concentration. In contrast to *Elovl1*, *Elovl7* was strikingly increased during cell differentiation. This suggests that ELOVL7 plays a crucial role in FA biosynthesis which, together with FA uptake, is required to allow the global increase in lipid content during oligodendrocyte differentiation and myelination [29]. Concordantly, *Elovl7* expression was drastically reduced in a mouse model of CNS hypomyelination [23]. Through inhibition of *Elovl7* expression, BPA might contribute to disrupt global FA synthesis. BPA could also influence the distribution of FA in phospholipids through a specific reduction in PUFA synthesis.

Overall, our results support that BPA disrupted the differentiation process in Oli-neu. Concordantly, BPA has previously been shown to inhibit the differentiation of primary mouse OPCs [30]. This effect was associated with a disturbance in thyroid hormone signaling and decreased thyroid hormone receptor (TRβ1) expression [30]. BPA has also been shown to decrease the myelination potential of rat neural stem cells [22]. In vivo, BPA exposure during gestation and lactation has been associated with myelin decompaction, g-ratio alteration, and decreased MBP, CNPase, PLP, and MAG levels in the hippocampus of adult rats [22]. Hypomyelination and oligodendrocyte loss in the hippocampus induced by early BPA exposure has also been associated with a deficit in contextual fear memory [31]. Together with previous in vitro data, our study evidenced a direct impact of BPA on early oligodendrocyte differentiation which, in turn, could impair myelination and, therefore, contribute to neurodevelopmental disorders. Finally, our results support that the Oli-neu cell line is a valuable model to screen chemicals for neurodevelopmental toxicity, especially through a lipidic composition analysis. It is noteworthy that the present study is based on an in vitro model mimicking early oligodendrocyte differentiation; assessment of mature myelin composition would require the implementation of glial-neuronal co-cultures or in vivo approaches.

## 5. Conclusions

In the present study, we highlighted a significant redistribution of lipids during differentiation in an oligodendroglial cell line. This redistribution involved characteristic lipids of myelin, especially ST and ePE. Changes in Oli-neu ST content were supported by the increased expression of genes associated with the sphingolipid biosynthetic pathways. The lipidome investigation, thus, appeared to be a valuable approach to study differentiation in oligodendrocytes and it was used to assess the impact of BPA on this process. In differentiated Oli-neu, BPA induced a decrease in ST and ePI contents, and a modification of MUFA/PUFA relative amounts in phospholipids. These changes counteract the lipid remodeling accompanying differentiation, suggesting that BPA disrupts the differentiation process in the Oli-neu lineage. Through an exhaustive analysis of lipids, the main constituents of myelin, this study provides a new perspective on the deleterious impact of BPA on oligodendrocyte differentiation. These results, therefore, contribute to a deeper understanding of the effects of BPA in the immature brain.

## Data Availability

Data supporting this article are given in the manuscript and in Supplementary Materials.

## Supplementary Materials

The following supporting information can be downloaded at: https://www.mdpi.com/article/10.3390/molecules27072274/s1, Supplementary Figure S1: Principal component analysis (PCA) models comparing lipid distribution (mol%) of proliferation P0 and differentiated groups D0, D1, and D10, Supplementary Table S1: qPCR primer sequences, Supplementary Table S2: Lipid distribution (mol%) in all samples, Supplementary Table S3: Significantly altered lipid species (*t*-test, FDR 5%) between P0 and D0 (P0-D0_significant_mol%) and between D0 and D10 (D0-D10_significant_mol%), Supplementary Table S4: Lipid subclass distribution (mol%) in each sample (Heatmap_Metaboanalyst) and each group (Lipidsubclass_means).
