# Supplementary material for: Bisphenol A Impairs Lipid Remodeling Accompanying Cell Differentiation in the Oligodendroglial Cell Line Oli-Neu
Source: Molecules. 2022 Mar 31;27(7):2274. doi: 10.3390/molecules27072274 (PMC9000593; doi:10.3390/molecules27072274)
Supplement: Supplementary file 1 [file molecules-27-02274-s001.zip › molecules-1622349-SI/Supplementary_materials_Figure 1+ Table 1.pdf]

Bisphenol A impairs lipid remodeling accompanying cell differentiation in the oligodendroglial cell line oli-neu

Naffaa Vanessa[1], Hochar Isabelle[1], Lama Chéryane[1], Magny Romain[1,2], Regazzetti Anne[1], Gressens Pierre[3], Laprévote Olivier[1,4], Auzeil Nicolas[1,#], Schang Anne-Laure[5,#,*]

#: contributed equally to this work

*Corresponding author: anne-laure.schang@parisdescartes.fr

[1] Université de Paris, CNRS, CiTCoM, F-75006 Paris, France

[2] Sorbonne Université, UM80, INSERM UMR 968, CNRS UMR 7210, Institut de la Vision, IHU ForeSight, Paris, France

[3] Université de Paris, NeuroDiderot, Inserm, F-75019 Paris, France

[4] Hôpital Européen Georges Pompidou, AP-HP, Service de Biochimie, Paris, France

[5] Université de Paris, UMR 1153 CRESS, Paris, France

Supplementary materials

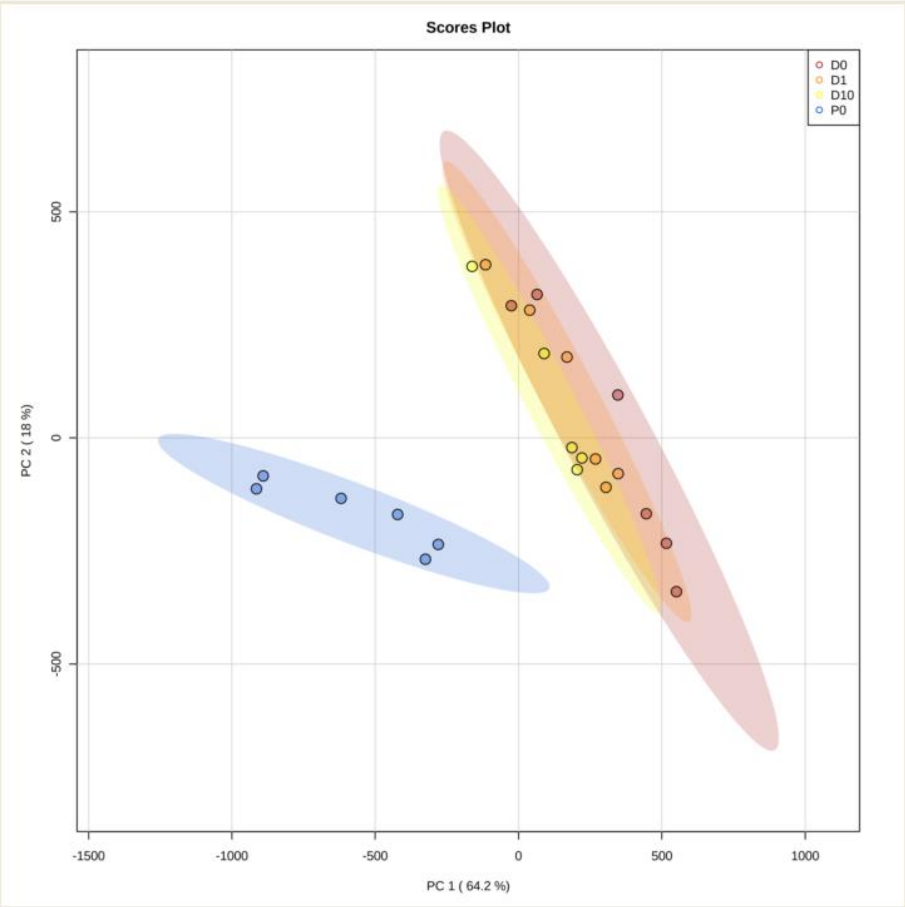

Supplementary Figure S1: Principal component analysis (PCA) models comparing lipid distribution (mol %) of proliferation P0 and differentiated groups D0, D1 and D10.

Supplementary Table S1: qPCR primer sequences

Gene name	Forward (5'>3')	Reverse (5'>3')
Arsa	GGTGTTGACATCAGCCCCTT	TGGATCTCGTCTGGGTAGGG
CNPase	AGAGACCTCCAGGTGTGCTG	GGCTCATTGGGAATAGGGGAG
Elovl 1	CAGCCCTACCTTTGGTGGAA	GTGAAGGTAGCCAGCTGATGT
Elovl 2	ACTACGGCCTGTCTGTGTTC	TTGGCTGGAGCCAGGAAATC
Elovl 4	ATCTCCTGTTCGTGTGGCTG	CCGGAAACCTTCCCAAGTCA
Elovl 5	TCGATGCGTCACTCAGTACC	GATACAGGTACAGCGTGCCA
Elovl 6	CCGAAGATCAGCCCCAATGA	CACCAAGCCCGAGACAAGAA
Elovl 7	TGGGACCAGCCTACCAGAAG	ACCGGCCAGAGTGTTTTCAT
Gal 3st 1	TCCAAGGCCAAGGGGTTACT	AAGTCTCCCAAGCGCTTCAT
Galc	GGCTTGGGAAACCTCACCAT	AAGGGGACAGATGGTTCCAG
Gba	GCTAGATGACCAACGCTTGC	CATCAAAGGGCCTGTCCAGT
Gba2	ACCCTCATGGTGTCCCTGAT	GGTCCCATAATGCCGACAGG
Mag	GTCTCTACCCGGGATTGTCA	TCTGAGCCCTCTGCCGATAA
MBP	CCGGACCCAAGATGAAAAC	CCCAGGTCTGAGTGGGAATA
Mog	AAGAGGCAGCAATGGAGTTG	GACCTGCAGGAGGATCGTAG
Plp1	CCAAATGACCTTCCACCTGT	CGAAGTTGTAAGTGGCAGCA
Sgms1	TTTTAACCGGGTGCAGTGGG	TGGGTGTCACAAAGAGAGCA
Sgms2	TCTCGTAGCGCATGAACACT	CTTGGGATGGAGGTGGTGT
Smpd1	AATTGGCCACATCCCTCCAG	TTGCAGCTGGGCATGAAGTA
Smpd2	TCCAGCCATGAAGCTCAACT	TGAGACAGCCAAAGGGGAAG
Sptlc1	ACCTGGAGCGACTGCTAAAAG	TCCTGCGTTGTATGATCCCA
Sptlc2	TGCCTGTCAGCAGCTCATAC	TGGTCCCAGCCATACAATGAG
Ugcg	CATTCAACTCAGGGGTGTCCA	CGTACAGCGCAGAAAACAGG
Ugt 8a	CAGGCAAAAGGCATGGGGAT	TGCAGTCCTCCATGAAGAAGA
